# The Effect of Exposure to Persistent Organic Pollutants on Metabolic Health among KOREAN Children during a 1-Year Follow-Up

**DOI:** 10.3390/ijerph13030270

**Published:** 2016-02-29

**Authors:** Hye Ah Lee, Su Hyun Park, Young Sun Hong, Eun Hee Ha, Hyesook Park

**Affiliations:** 1 Department of Preventive Medicine, School of Medicine, Ewha Womans University, 1071, Anyangcheon-ro, Yangcheon-ku, Seoul 158-710, Korea; khyeah@naver.com (H.A.L.); shp323@ewha.ac.kr (S.H.P.); eunheeha@ewha.ac.kr (E.H.H.); 2 Department of Internal Medicine, School of Medicine, Ewha Womans University, 1071, Anyangcheon-ro, Yangcheon-ku, Seoul 158-710, Korea; imhys@ewha.ac.kr

**Keywords:** cohort study, children, metabolic syndrome, persistent organic pollutants

## Abstract

Previous evidence suggests the potential for adverse effects of persistent organic pollutants (POPs) on metabolic health even at low-dose exposure levels common among the general population, but there is less evidence of these associations among children. Therefore, as part of a prospective cohort study, 214 children were measured for POPs exposure. After the 1-year follow-up, we assessed the effect of circulating POPs exposure among 158 children aged 7–9 years (at baseline) on the change of metabolic components of metabolic syndrome using multiple regression analysis. In addition, we calculated the continuous metabolic syndrome (cMetS) score and assessed the variation among individuals by POPs exposure. The concentrations of marker polychlorinated biphenyls (PCBs) were significantly associated with increased change in diastolic blood pressure (BP) and triglyceride levels during a 1-year follow-up, after controlling for sex, age, household income, and change in body mass index. Total PCBs also showed a marginal association with increasing cMetS score from the baseline. Of the metabolic components, change in diastolic BP over time showed a notable association with specific PCBs, but no association with organochlorine pesticides. Here, we found that low-dose exposures to PCBs among children in the general population could negatively influence metabolic health, particularly diastolic BP. Increased disease sensitivity during childhood can continue to adulthood, thus, these results support the need for continuous assessment of the health impact of POPs.

## 1. Introduction

Metabolic syndrome (MetS) represents a cluster of metabolic abnormalities commonly defined by obesity, glycosemia, reduced high-density lipoprotein cholesterol (HDL-c) level, hypertriglyceridemia, and hypertension [1,2]. In addition, these are known precursors to cardiovascular disease.

Mounting evidence from experimental and epidemiological studies suggests that exposure to endocrine disruptors such as persistent organic pollutants (POPs) may be linked to metabolic dysfunction, such as obesity [3], insulin resistance [3], dyslipidemia [3,4], hypertension [5,6,7,8,9], and MetS morbidity [10] or new-diagnosed MetS [11].

POPs (including PCBs and OCPs) are synthetic (artificially produced) organic chemicals used in pesticides or industrial products. POPs can accumulate in the food chain and tend to remain in fat-rich tissues. The impact of POP accumulation on the lifespan remains unclear, but it has been suggested that POPs function as endocrine disruptors.

The increased concerns regarding the health risks of endocrine disruptors, including POPs, has resulted in legal limitations of their use [12]. Nonetheless, the harmful impact of low-dose endocrine disruptor exposure on health remains problematic [3,9,11]. It is known that children are more likely to metabolize endocrine disruptors and are therefore more vulnerable to their harmful effects than adults [13]. An epidemiological study conducted among 318 Russian boys residing in a high-risk region showed that circulating organochlorine pesticides (OCPs) were associated with insulin resistance after a 4 year follow-up, with no association in lipid indicators [14]. However, the effects of POPs on the metabolic health of children remain unclear, especially in terms of the exposure levels in general populations.

In general, the prevalence of MetS is below 5% in children and adolescents [2,15]. However, due to the lack of a universal definition of MetS among children and adolescents, there are variations in the prevalence of MetS even within the same data sources [1]. The low prevalence and inconsistent definition represent challenges to studying metabolic diseases among children, especially in studies with small sample sizes. For these reasons, several epidemiological studies have been studied by creating a continuous metabolic syndrome (cMetS) score representing a composite metabolic health profile [16,17,18]. It was argued that the cMetS score has sufficient statistical strength, with less error and greater sensitivity, than the traditional definition of MetS, which is defined as dichotomous [1].

Using a prospective approach, which can demonstrate temporal relationships, we conducted this study to answer the following two questions: (1) Are common POP exposure levels among Korean children associated with change of metabolic susceptibility, as assessed by cMetS score?; and (2) Which metabolic components are most sensitive to common POP exposure levels among children?

## 2. Methods

### 2.1. Study Subjects

This study was conducted as a part of the Ewha Birth & Growth Cohort study. The Ewha Birth & Growth Cohort study was initiated in 2001 among pregnant women who sought prenatal care at the Ewha Womans Mokdong Hospital, which is a tertiary hospital located in southwest Seoul, Korea. This cohort study was established to focus on child growth and health with longitudinal observation beginning in early life. From 2001 to 2006, a total of 940 pregnant women enrolled in the study. Details regarding the cohort composition and methodology have been previously published elsewhere [19].

This cohort was not initially intended to assess environmental exposures, and measuring POPs was an additional cost. In addition, there was limited blood available from children. In general, puberty occurs after 10 years of age. Thus, we consider that all participants were pre-pubertal. We selected 214 children aged 7 to 9 years (boys, 106; girls, 108) at baseline, 158 of whom (boys, 82; girls, 76) completed the 1-year follow-up. At baseline and follow-up, we collected anthropometric measurements, socioeconomic data (by questionnaire), and blood samples. Blood samples were obtained from an antecubital vein into vacutainer tubes containing EDTA after an overnight fasting period. All blood samples obtained from the subjects were stored at −70 °C.

All participants gave their informed consent for study participation. The study was approved by the Institutional Review Board of the Ewha Womans University Hospital (ECT 13-10A-30).

### 2.2. Persistent Organic Pollutants Analysis

At baseline, samples from 214 children were measured for 32 polychlorinated biphenyls (PCBs) (IUPAC numbers: 1, 3, 4, 15, 19, 28, 37, 52, 54, 77, 81, 101, 104, 105, 114, 118, 123, 126, 138, 153, 155, 156, 157, 167, 169, 180, 188, 189, 202, 205, 206, and 208) and 19 OCPs—(oxychlordane, (*trans-*, *cis-*) nonachlor, (*trans-*, *cis-*) chlordane, heptachlor, (*trans-*, *cis-*) heptachlor epoxide, hexachlorobenzene (HCB), α-hexachlorocyclohexane (α-HCH), β-HCH, γ-HCH, δ-HCH, *p*,*p’*-dichlorodiphenyltrichloroethane (*p*,*p’*-DDT), *o*,*p’*-DDT, *p*,*p’*-dichlorodiphenyldichloroethane (*p*,*p’*-DDD), *o*,*p’*-DDD, *p*,*p’*-dichlorodiphenyldichloroethylene (*p*,*p’*-DDE), and *o*,*p’*-DDE)—using an isotope dilution method with gas chromatography high–resolution mass spectrometry (GC/HRMS). The coefficient of variance (CV) of quality assurance/quality control (QA/QC) was <15% for all compounds when the values were above the limits of detection (LOD) and a detailed description of the measurement methods has been published previously [20].

Of the compounds measured, seven PCBs (52, 101, 118, 138, 153, 156, and 180) and HCB, *trans*-Nonachlor, β-HCH, *p*,*p’*-DDT, and *p*,*p’*-DDE were measured in more than 60% of the study subjects. Thus, the statistical analysis of specific PCBs and OCPs was conducted on the above list. Several congeners were classified according to common action, such as dioxin-like PCBs (77, 81, 105, 114, 118, 123, 126, 156, 157, 167, 169, and 189). The variable “total PCBs” included 32 PCBs (ΣPCBs) and the 6 marker PCBs congeners (28, 52, 101, 138, 153, and 180) were also considered. Below the LOD was imputed using the equation: LOD/√2. The LODs of the POPs studied have been reported previously [20].

### 2.3. Metabolic Components and Continuous Metabolic Syndrome (cMetS) Score

The metabolic components considered were body mass index (BMI), glucose, HDL-c, triglyceride (TG), and mean arterial blood pressure (MAP). Height and weight were measured with no shoes and light clothing using a stadiometer and a calibrated scale (DS-102, Dong Sahn Jenix Co. Ltd., Seoul, Korea). Systolic and diastolic blood pressure (BP) were measured twice by trained researchers using an automatic device (Critikon, Tampa, FL, USA) with the correct cuff size after participants rested in a stable position. Two measurements, taken within 5 min of each other, were averaged. The MAP was calculated using the formula: [(systolic BP − diastolic BP)/3] + diastolic BP. Blood glucose, HDL-c, and TG were measured with an automatic analyzer (model 7180; Hitachi, Tokyo, Japan). These components were measured both at baseline and at follow-up.

To calculate the cMetS score, we used the z score method. For each of five metabolic components, standardized z scores were calculated using the formulation: (individual value—mean)/standard deviation. Means (with standard deviations) were derived using the combined data from the entire Ewha Birth & Growth Cohort, stratified by age. Because HDL-c is known to have an inverse association with MetS, the standardized value for HDL-c was multiplied by –1. Then, the standardized z-scores for BMI, glucose, TG, MAP, and HDL-c were summed for each subject. Higher cMetS scores indicate an unfavorable MetS status.

### 2.4. Statistical Analysis

In the descriptive analysis, results are shown as medians with inter quartile range for skewed distributed data and as means with standard deviation for normal data. Normally distributed numerical variables were analyzed using the Kolmogorov-Smirnov Test. The degree of correlation for repeated measures of metabolic components was analyzed using the Pearson correlation. To account for the baseline effects across individuals, an individual relative change (%) was calculated for the metabolic components and cMetS scores were represented as differences (∆) over time.

In the regression model analysis, the specific POPs on a lipid adjustment scale did not satisfy the assumption of normality, so these values were transformed into natural logarithm values. The change of metabolic components including cMetS score across the specific POPs and the three exposure groups (Dioxin-like PCBs, marker PCBs, and ΣPCBs) were assessed using multiple linear regression. As potential confounding variables, we considered sex (boys and girls), age (months), monthly household income (low: <3 million KRW, middle: 3–4.9 million KRW, and high: ≥5 million KRW, KRW: South Korean Won), and BMI at baseline. Additionally, we considered the change in BMI during a 1-year follow-up period; BMI and BMI change were selected based on previous studies [4,9]. Socioeconomic status factors included maternal education level (low: graduated high school, high: some college or higher), monthly household income, and maternal occupation (Yes or No). In the univariate analysis, monthly household income showed a marginal association with dioxin-like PCBs (*p* = 0.07) concentration, and so it was included as a covariate.

For sensitivity analysis, to assess non-linear associations, tertiles of specific POPs were analyzed using general linear models and trend tests, controlling for sex, age, household income, and BMI at baseline. A *p* for trend of <0.05 was considered significant for linear association. Without regard to the effects of BMI, we analyzed the effect of POPs on the change in metabolic components adjusted only for sex, age, and monthly household income. In addition, we re-analyzed the multiple regression using wet-weight concentration of POPs. All analyses were conducted using SAS software (ver. 9.3; SAS Institutes, Cary, NC, USA). A *p* value of <0.05 was considered to be statistically significant under the two-tailed test.

## 3. Results

The wet-weight concentrations and lipid-adjusted concentrations for study subjects are given in Table 1. Of 214 children, 108 (50.5%) were girls and the mean age was 90.1 ± 9.5 months. Monthly household income levels were 17.6% low, 41.4% middle, and 41.0% high. The proportion of children with mothers who worked was 36.8%, and low maternal education was seen in 21.3% of cases at baseline. Those with higher household incomes showed higher concentrations of dioxin-like PCBs (low: 2.34 ng/g lipid, middle: 2.57 ng/g lipid, and high: 3.14 ng/g lipid, *p* = 0.07), while other socioeconomic factors were not associated with POP concentrations (data not shown).

Averages of metabolic components at baseline, and changes in metabolic components during a 1-year follow-up, are presented in Table 2. At the 1-year follow-up, the relative changes in metabolic components were +4.78% for BMI, +2.05% for glucose, −3.06% for HDL-c, +3.84% for TG, +3.66% for systolic BP, and +6.43% for diastolic BP. The mean cMetS score was −0.32 at baseline and increased by 0.08 after one year. The differences in metabolic components at baseline among the follow-up subjects (*n* = 158) and subjects lost to follow-up (*n* = 56) were not significant except for glucose (follow-up subjects: 76.2 mg/dL, subjects lost to follow-up: 80.5 mg/dL, *p* = 0.001).

The correlation of metabolic components for two repeated measurements over one year was highest for BMI (*r* = 0.93) and lowest for glucose (*r* = 0.30). The cMetS score also showed a moderate positive correlation (*r* = 0.62, *p* <0.0001) (Table 2).

Based on the multiple regression analysis adjusted for sex, age, monthly household income, and BMI at baseline, the cMetS score increased over 1-year follow-up time by 0.63 units for every 1 unit increase in baseline concentration of PCB 52, which corresponds to e^1^ (one exponential unit) = 2.*7*. The other differences did not attain statistical significance. Diastolic BP increased by an average of 2.5% and 3.4%, respectively, for each 2.7-fold increase in PCBs 138 and 153 on an ng/g lipid scale. Diastolic BP increased by around 5% with each one unit increase in log transformed ΣPCBs and marker PCBs. Dioxin-like PCBs also showed a positive change with diastolic BP, but it did not reached statistical significance (Table 3).

The results adjusted for change of BMI over time, instead of for BMI at baseline, are shown in Table 4. Similar tendencies were seen for relationships between change in metabolic components and specific PCBs, ΣPCBs, and marker PCBs. Both ΣPCBs and marker PCBs were notably related to variation in cMetS score when adjusting for change in BMI over time, although these showed a borderline significance.

Contrary to expectations, change in glucose and HDL-c showed an inverse association with specific POPs. In addition, no relationships between OCPs and change in metabolic components or cMetS score were found regardless of adjustment for BMI at baseline or BMI change over time.

Regarding non-linear associations, tertiles of PCBs were evaluated and the linear association still remained. In the absence of adjustment for BMI or BMI change score, the results of the effects of PCB 52 on variation in cMetS score and the effects of PCBs 138, 153, ΣPCBs, and marker PCBs on change of diastolic BP over time did not change significantly.

When we analyzed data using wet-weight POP concentrations, PCBs 138 and 153, ΣPCBs, and marker PCBs still showed a positive association with change in diastolic BP over time after controlling for sex, age, household income, total lipid concentration at baseline, and BMI at baseline or change in BMI over follow-up time. However, although a significant association between variation in cMetS score and PCB 52 remained, the relationships with ΣPCBs and marker PCBs were statistically insignificant.

## 4. Discussion

In this study, marker PCBs and ΣPCBs were associated with elevated diastolic BP and dioxin-like PCBs showed a marginal association. In particular, PCB 52 of marker PCBs showed unfavorable effects on change in cMetS scores, even after adjusting for potential confounders. Background exposure to PCBs was notably associated with change in diastolic BP. In addition, we found that the association with individual cMetS score persisted during the 1-year follow-up, and showed a moderate positive correlation (*r* = 0.62). To the best of our knowledge, this is the first prospective study of the effects of POP exposure on metabolic health risk using cMetS score among children, even if was a short-term assessment.

Although there are regulations for preventing exposure to POPs [12] several PCBs congeners and OCPs were still measured in the blood. According to national monitoring projects, the levels of PCBs in water and soil, as well as marine deposition in the area, were lower than the domestic environmental standards [12]. In addition, measureable levels of PCBs were found in fish and meat, but were also lower in comparison to the levels found in Japan, the US, and Germany, accounting for 0.3% of the 5 μg/kg body weight/day tolerable daily intake [12]. However, low-dose persistent exposure can affect health and developing children could be more vulnerable [13,14]. Childhood is a time of various physical and endocrine changes, and is also likely to be a period of greater sensitivity to endocrine disrupters [13]. However, there is a lack of studies on the impact of endocrine disruptors on children. The impact of POPs on lifespan, and the mechanisms by which they impact disease, are not clear, but it is suggested that they play a part in the inflammation response, disruption of endocrine metabolism, and adipocyte function [21]. In addition, studies conducted in adults have also found associations of exposure to endocrine disruptors with reproductive health and metabolic disease [22]. Although previous studies did not demonstrate causality with respect to the effect of POPs on health risk, the above bioactivities could be related to metabolic dysfunction.

In a study focusing on MetS in the general population, quartiles of dioxin-like PCBs, non-dioxin-like PCBs, and OCPs showed a significant association with the prevalence of MetS [10], and a nested case-control study in Korean community-based study showed summary measures of 15 PCBs associated with MetS [11]. Although both studies cited above were conducted in adults, the age distributions of the subjects differed. In addition, a later study found a non-linear association whereas our data and those of Lee *et al.* [10] suggest that the associations are linear. To the best of our knowledge, there has been no study of MetS among children focusing on normal background exposure to POPs. Thus, the dose relationships between POP levels and MetS in children require further evaluation after more studies have been conducted. In the present study, of the six marker PCB congeners examined, the relationship between PCB 52 and cMetS score was especially notable. PCB 28 and 52 contaminate indoor air, whereas the others commonly contaminate food [23]. Thus, our results suggest strategies for reducing exposure and improving health policies.

Several other studies showed a moderate positive correlation in changing cMetS score from childhood to young adulthood, such as the Bogalusa Heart study with eight years of follow-up (tracking correlation (*r*) = 0.54) [17], and the Quebec Family study with 12 years of follow-up (*r* = 0.51 in male and *r* = 0.46 in female) [18], although there were differences in calculation method for cMetS scores. Similarly, our results showed a moderate correlation (*r* = 0.62). We thought that individual cMetS score, which was calculated as the sum of metabolic component z scores at baseline and 1-year follow-up, sufficiently indicated the overall individual metabolic state compared with the average for each age. Thus, variation in cMetS score may reflect changes in individual metabolic state. One large prospective study also reported that cMetS z score predicts diagnosis of MetS and suggested tracking MetS within individuals over time [16]. However, as yet there is no consensus on how to calculate the cMetS score.

Mounting evidence from prospective epidemiological studies accounting for temporality showed an association between specific PCBs and higher BMI, higher TG, higher HOMA-IR [3], lower HDL-c [3], and metabolic syndrome [11]. Additionally, specific OCPs also showed an association with insulin resistance [3,14] and dyslipidemia [3,4]. In our study, an increase of the exposure level to specific PCBs by one unit at baseline appeared to be associated with a positive relative change in diastolic BP and TG. These linear associations remain when evaluating POP exposure as tertiles (*p* for trend <0.05, data not shown). Among the individual metabolic components, our results showed a notable association with change in diastolic BP. With regard to hypertension, a series of studies conducted in Anniston, Alabama showed that serum PCBs, notable in multiple *ortho* chlorines, had a strong positive association with both high systolic and diastolic BP in those with normal-range BP [7,8]. In contrast, there was no association between OCPs and BP, in line with previous studies [8,9,10]. Experimental studies have reported that dioxin-like PCBs induced elevated blood pressure and explained this biological mechanism as being closely related to aryl hydrocarbon receptor (AhR) activation [5,24]. Although accumulating evidence shows a possible association between POPs and metabolic health risk, underlying mechanisms for the impact of POPs on health in humans are not clear.

It is well known that lipophilic POPs accumulate in the adipocyte and have long half-lives, leading to slow elimination from the body [9]. In addition, the range of circulating concentrations of POPs varied by subject, according to differences in age, race, geography, and socioeconomic status. Several studies have been conducted among residents in high-risk areas for POP exposure [8,14]. In our study, those with a high income showed higher concentrations of dioxin-like PCBs. A study by Ha *et al.* [9] also reported that PCB concentrations were higher in those with higher incomes. Compared to other studies focusing on children, the ranges of OCP concentrations noted in our present study were lower than those in a study performed in boys in Chapaevsk, Russia [14]. The average concentrations of PCBs 153 and 180 in this study were lower than in a Danish study and higher than in a Chinese study [25]. Inconsistent findings may result from these differences in exposure range. Additionally, there are issues for validity of lipid adjustments, adjustment method, and BMI adjustments due to the lipophilic character of POPs [26]. However, several studies, including this study, did not show substantial changes in results after lipid adjustment [9]. When adjusting for BMI change instead of baseline BMI, the effects of ΣPCBs and marker PCBs were more significantly associated with variation in cMetS score. In addition, the results did not change after excluding BMI as a covariate.

Generally speaking, one of the major routes suggested for exposure to POPs is food intake. These results did not take into account any dietary effects, so that potential confounder remains, thereby limiting the interpretation of our results. Residual effects that we did not consider (e.g., the BMI of the mother or both parents, the level of physical activity, the consumption levels of sugared beverages) could have influenced our results, but studies in children will be less affected by the confounding effects of unhealthy behaviors associated with metabolic health (e.g., alcohol consumption) than are those in adult studies. Although the glucose levels of those lost to follow-up were significantly higher than those of subjects who were followed up, our data were not significantly affected by selection bias, as the levels of other metabolic components did not differ between the two subsets of subjects. Finally, there is an issue with respect to generalization, due to the small sample size.

This study used cMetS score instead of MetS diagnosis to increase statistical power, given the small sample size. This allowed us to avoid misclassification bias, but there may have been measurement error. Some studies have argued that cMetS score has greater statistical power, but it has limitation in comparison with other studies [1]. Nevertheless, we confirmed the possibility of a temporal relationship and our results were in line with previous studies. Our study increases the evidence of a potential association between POPs and metabolic health risk among children in the general population. In addition, further studies are required to continuously evaluate the metabolic health risks posed by POPs; long-term follow-up is essential.

## 5. Conclusions

In this study, we found that normal background exposure to PCBs among children in the general population could negatively influence metabolic health in terms of changes in the metabolic components of MetS and cMetS score over time. Children in developmental stages who are exposed to POPs could be more vulnerable than adults, and any exposure could affect their health in later life. Thus these results support the need for continuous health impact assessment.

## Figures and Tables

**Table 1:** Descriptive analysis of concentrations of circulating persistent organic pollutants in children aged 7 to 9 years at baseline (*N* = 214).

Persistent Organic Pollutants	Detection Rate (%)	Lipid Adjusted Concentration (ng/g Lipid)			Wet-Weight Concentration (pg/mL)		
		Median	25th Percentile	75th Percentile	Median	25th Percentile	75th Percentile
PCB 52	92.52	2.54	1.50	3.53	15.08	8.70	19.61
PCB 101	89.72	1.15	0.76	1.58	6.53	4.79	8.98
PCB 118	96.26	1.56	1.07	2.12	8.95	6.27	11.49
PCB 138	99.07	2.32	1.60	3.61	13.30	9.48	21.02
PCB 153	99.07	4.27	2.70	7.49	24.27	15.92	41.43
PCB 156	73.83	0.57	0.21	0.98	3.30	0.21	5.62
PCB 180	98.13	2.56	1.65	4.90	16.27	10.10	29.98
Σ PCB		24.63	14.58	35.29	143.47	91.11	208.81
Marker PCB		16.78	10.41	24.41	94.10	63.49	137.97
Dioxin-like PCB		3.01	1.64	4.60	17.54	10.08	26.99
HCB	70.09	5.65	0.60	18.58	33.10	0.60	103.82
*trans*-Nonachlor	61.68	1.19	0.64	2.05	6.56	0.64	11.53
β-HCH	96.26	6.13	3.80	11.19	36.30	21.81	62.40
*p*,*p’*-DDT	99.07	3.03	2.31	4.04	17.65	13.49	23.61
*p*,*p’*-DDE	99.53	43.46	26.74	71.24	249.61	159.82	406.75

PCB, polychlorinated biphenyl; HCB, hexachlorobenzene; β-HCH, β-hexachlorocyclohexane; *p*,*p’*-DDT, *p*,*p’*-dichlorodiphenyltrichloroethane; *p*,*p’*-DDE, *p*,*p’*-dichlorodiphenyldichloroethylene; Results below the limit of detection (LOD) were imputed using the equation: LOD/√2. ΣPCB indicates the sum of the levels of all 32 PCB congeners. Marker PCB indicates the sum of the levels of PCBs 28, 52, 101, 138, 153, and 180. Dioxin-like PCB indicates the sum of the levels of PCBs 77, 81, 114, 105, 118, 126, 123, 156, 157, 169, 167, and 189.

**Table 2:** Variation in change in metabolic components and continuous metabolic syndrome score during the 1-year follow-up.

Outcomes	Mean	SD	95% CI		Correlation	
			Lower	Upper	*r*	*p* Value
cMetS ^a^	−0.32	2.90	−0.72	0.08	0.62	<0.0001
∆ cMetS	0.082	2.45	−0.30	0.47		
BMI (kg/m^2^) ^a^	16.75	2.48	16.41	17.09	0.93	<0.0001
Relative change of BMI (%)	4.78	5.63	3.90	5.66		
Fasting Glucose (mg/dL) ^a^	79.48	7.82	78.42	80.55	0.30	0.0001
Relative change of glucose (%)	2.05	13.16	−0.03	4.12		
HDL-c (mg/dL) ^a^	61.44	11.66	59.85	63.03	0.75	<0.0001
Relative change of HDL-c (%)	−3.06	13.27	−5.15	−0.97		
TG (mg/dL) ^a^	57.99		53.97	62.31	0.39	<0.0001
Relative change of TG (%)	3.84	14.15	1.61	6.07		
MAP (mmHg) ^a^	73.69	7.07	72.72	74.66	0.60	<0.0001
Relative change of MAP (%)	4.97	9.75	3.44	6.50		
SBP (mmHg) ^a^	100.83	9.12	99.59	102.08	0.57	<0.0001
Relative change of SBP (%)	3.66	10.12	2.07	5.25		
DBP (mmHg) ^a^	60.12	8.32	58.98	61.25	0.50	<0.0001
Relative change of DBP (%)	6.43	14.08	4.22	8.64		

cMetS, continuous metabolic syndrome; BMI, body mass index; HDL-c, high-density lipoprotein cholesterol; TG, triglyceride; MAP, mean arterial blood pressure; SBP, systolic blood pressure; DBP, diastolic blood pressure; SD, standard deviation; 95% CI, 95% confidence interval. Triglyceride values did not follow a normal distribution and so were analyzed as natural log transformed values, with results presented as back-transformed values. ^a^ Results at baseline (*N* = 214); the variation results were estimated from 158 children who participated during the 1-year follow-up. Correlation coefficients for repeated measures of metabolic components were obtained from the Pearson correlation. Mean arterial blood pressure (MAP) was calculated as: [(systolic BP − diastolic BP)/3] + diastolic BP. Relative change (%) was calculated as: (values at follow-up − values at baseline)/values at baseline × 100. Continuous metabolic syndrome (cMetS) score was evaluated as difference (∆) over time.

**Table 3:** The effect of circulating lipid-adjusted persistent organic pollutants (natural logarithms value) on the change in metabolic components and continuous metabolic syndrome score over 1-year follow-up, controlling for baseline body mass index.

Persistent Organic Pollutants	∆ cMetS Score			Relative Change of BMI (%)			Relative Change of Glucose (%)			Relative Change of HDL−c (%)			Relative Change of TG (%)			Relative Change of SBP (%)			Relative Change of DBP (%)		
	*β*	95% CI		*β*	95% CI		*β*	95% CI		*β*	95% CI		*β*	95% CI		*β*	95% CI		*β*	95% CI	
PCB 52	**0.63a**	**0.18**	**1.07**	0.03	−1.03	1.10	1.83	−0.54	4.21	−0.30	−2.84	2.23	2.53	−0.07	5.14	0.44	−1.46	2.35	2.09	−0.53	4.70
PCB 101	0.38	−0.06	0.82	−0.01	−1.04	1.02	−0.20	−2.52	2.12	0.02	−2.43	2.47	1.41	−1.13	3.96	0.07	−1.77	1.92	0.88	−1.67	3.42
PCB 118	0.36	−0.36	1.09	−0.29	−1.84	1.27	0.33	−3.44	4.09	−0.57	−4.56	3.42	0.75	−3.40	4.90	−0.12	−3.12	2.87	3.56	−0.55	7.66
PCB 138	0.24	−0.28	0.76	0.14	−1.02	1.29	−0.98	−3.69	1.74	−0.55	−3.42	2.33	−0.04	−3.04	2.95	0.72	−1.42	2.87	**3.38a**	**0.45**	**6.30**
PCB 153	0.03	−0.36	0.42	−0.22	−1.09	0.64	−0.10	−2.12	1.93	0.65	−1.49	2.79	−0.44	−2.67	1.79	0.87	−0.73	2.48	**2.52a**	**0.33**	**4.71**
PCB 156	0.17	−0.40	0.73	0.22	−1.05	1.50	−2.20	−5.13	0.72	−2.39	5.49	0.70	0.12	−3.12	3.37	1.50	−0.83	3.82	3.02	−0.18	6.21
PCB 180	0.14	−0.27	0.56	−0.13	−1.05	0.79	0.75	−1.40	2.90	−0.88	−3.15	1.39	0.81	−1.56	3.18	1.35	−0.35	3.05	2.27	−0.07	4.61
ΣPCB	0.49	−0.22	1.19	0.39	−1.16	1.94	−1.58	−5.20	2.04	−1.77	−5.70	2.17	2.93	−1.13	6.99	1.33	−1.61	4.26	**5.70a**	**1.75**	**9.66**
Marker PCB	0.39	−0.28	1.06	0.30	−1.18	1.79	−1.70	−5.14	1.75	−1.08	−4.83	2.66	3.04	−0.81	6.90	1.65	−1.14	4.44	**5.12a**	**1.34**	**8.90**
Dioxin-like PCB	−0.08	−0.73	0.57	−0.03	−1.45	1.40	**−3.28a**	**−6.57**	**0.00**	−1.30	−4.90	2.31	−0.09	−3.83	3.65	0.66	−2.03	3.35	3.44	−0.23	7.11
HCB	0.09	−0.15	0.34	0.07	−0.48	0.62	**−1.52a**	**−2.79**	**−0.25**	0.31	−1.05	1.68	0.84	−0.57	2.26	0.17	−0.86	1.20	0.59	−0.83	2.01
*trans*-Nonachlor	−0.19	−0.75	0.37	0.35	−0.88	1.58	−1.46	−4.38	1.47	0.76	−2.34	3.87	−0.58	−3.81	2.65	−0.29	−2.61	2.03	2.21	−0.98	5.39
β-HCH	−0.26	−0.59	0.08	−0.50	−1.26	0.27	0.43	−1.34	2.20	1.85	0.00	3.70	−1.66	−3.59	0.27	0.00	−1.41	1.41	1.04	−0.89	2.98
*p,p’*-DDT	−0.26	−0.91	0.40	−0.83	−2.27	0.61	0.74	−2.67	4.14	1.84	−1.75	5.43	−1.64	−5.38	2.11	−0.59	−3.30	2.12	1.83	−1.91	5.57
*p,p’*-DDE	−0.17	−0.58	0.24	−0.51	−1.42	0.41	0.64	−1.50	2.77	1.91	−0.33	4.16	−1.65	−4.00	0.69	0.08	−1.62	1.78	1.71	−0.63	4.04

^a^ *p* < 0.05; cMetS, continuous metabolic syndrome; BMI, body mass index; HDL-c, high-density lipoprotein cholesterol; TG, triglyceride; SBP, systolic blood pressure; DBP, diastolic blood pressure; PCB, polychlorinated biphenyl; HCB, hexachlorobenzene; β-HCH, β-hexachlorocyclohexane; *p,p’*-DDT*, p,p’*-dichlorodiphenyltrichloroethane; *p,p’*-DDE, *p,p’*-dichlorodiphenyldichloroethylene. Results below the limit of detection (LOD) were imputed using the equation: LOD/√2. Multiple regression models are adjusted for sex, age (months), household income, and BMI at baseline. The regression model for change in BMI is adjusted for sex, age (months), and household income. Relative change (%) in the metabolic components was calculated as: (values at follow-up − values at baseline) / values at baseline × 100. Continuous metabolic syndrome (cMetS) score was evaluated as difference (∆) over time. ΣPCB indicates the sum of the levels of all 32 PCB congeners. Marker PCB indicates the sum of the levels of PCBs 28, 52, 101, 138, 153, and 180. Dioxin-like PCB indicates the sum of the levels of PCBs 77, 81, 114, 105, 118, 126, 123, 156, 157, 169, 167, and 189.

**Table 4:** The effect of circulating lipid-adjusted persistent organic pollutants (natural logarithms value) on the change in metabolic components and continuous metabolic syndrome score over the 1-year follow-up, controlling for change in body mass index over time.

Persistent Organic Pollutants	∆ cMetS Score			Relative Change of Glucose (%)			Relative Change of HDL−c (%)			Relative Change of TG (%)			Relative Change of SBP (%)			Relative Change of DBP (%)		
	*β*	95% CI		*β*	95% CI		*β*	95% CI		*β*	95% CI		*β*	95% CI		*β*	95% CI	
PCB 52	**0.64 ^a^**	**0.21**	**1.07**	1.91	−0.46	4.29	−0.21	−2.74	2.33	2.66	0.05	5.26	0.45	−1.42	2.32	2.05	−0.55	4.65
PCB 101	0.40	−0.03	0.82	−0.13	−2.46	2.19	0.09	−2.36	2.54	1.52	−1.03	4.07	0.09	−1.72	1.90	0.86	−1.68	3.40
PCB 118	0.56	−0.08	1.20	1.06	−2.45	4.56	0.20	−3.50	3.91	2.01	−1.84	5.86	0.15	−2.58	2.89	2.93	−0.87	6.74
PCB 138	0.31	−0.17	0.80	−0.50	−3.13	2.12	−0.08	−2.85	2.70	0.59	−2.30	3.48	0.71	−1.32	2.75	**3.01 ^a^**	**0.20**	**5.82**
PCB 153	0.14	−0.23	0.50	0.21	−1.76	2.18	0.86	−1.21	2.93	0.14	−2.03	2.31	0.95	−0.57	2.48	**2.33 ^a^**	**0.21**	**4.45**
PCB 156	0.22	−0.31	0.76	−1.77	−4.64	1.11	−1.90	−4.93	1.14	0.63	−2.55	3.82	1.43	−0.80	3.67	2.76	0.35	5.88
PCB 180	0.25	−0.14	0.63	1.03	−1.04	3.10	−0.52	−2.71	1.67	1.35	−0.93	3.63	1.36	−0.25	2.97	2.05	−0.20	4.30
ΣPCB	0.60	−0.04	1.24	−0.58	−4.02	2.85	−0.84	−4.55	2.87	3.66	−0.15	7.47	1.22	−1.49	3.93	**4.84 ^a^**	**1.11**	**8.57**
Marker PCB	0.51	−0.11	1.12	−0.81	−4.10	2.49	−0.34	−3.91	3.22	**3.71 ^a^**	**0.05**	**7.36**	1.54	−1.06	4.15	**4.44 ^a^**	**0.85**	**8.03**
Dioxin-like PCB	0.09	−0.50	0.68	−2.24	−5.39	0.91	−0.49	−3.91	2.93	0.72	−2.81	4.25	0.72	−1.78	3.23	3.01	−0.46	6.48
HCB	0.13	−0.10	0.36	−1.22	−2.46	0.01	0.49	−0.83	1.81	1.09	−0.27	2.46	0.18	−0.80	1.15	0.49	−0.88	1.85
*trans*-Nonachlor	−0.09	−0.61	0.43	−0.84	−3.65	1.96	1.25	−1.71	4.20	0.19	−2.90	3.28	−0.26	−2.43	1.91	1.83	−1.20	4.86
β-HCH	−0.13	−0.45	0.19	0.65	−1.10	2.39	**1.91 ^a^**	**0.09**	**3.74**	−1.13	−3.05	0.79	0.20	−1.16	1.57	1.05	−0.86	2.95
*p,p’*-DDT	0.03	−0.58	0.64	1.28	−1.99	4.56	2.11	−1.34	5.56	−0.31	−3.93	3.30	−0.15	−2.72	2.41	1.70	−1.89	5.28
*p,p’*-DDE	−0.01	−0.40	0.37	0.94	−1.14	3.01	2.03	−0.15	4.20	−0.88	−3.17	1.41	0.30	−1.31	1.92	1.61	−0.64	3.87

^a^ *p* < 0.05; cMetS, continuous metabolic syndrome; BMI, body mass index; HDL-c, high-density lipoprotein cholesterol; TG, triglyceride; SBP, systolic blood pressure; DBP, diastolic blood pressure; PCB, polychlorinated biphenyl; HCB, hexachlorobenzene; β-HCH, β-hexachlorocyclohexane; *p,p’*-DDT*, p,p’*-dichlorodiphenyltrichloroethane; *p,p’*-DDE, *p,p’*-dichlorodiphenyldichloroethylene. Results below the limit of detection (LOD) were imputed using the equation: LOD/√2. Multiple regression models are adjusted for sex, age (months), household income, and change in body mass index over time. Relative change (%) in the metabolic components was calculated as: (values at follow-up − values at baseline)/values at baseline × 100. Continuous metabolic syndrome (cMetS) score was evaluated as difference (∆) over time. ΣPCB indicates the sum of the levels of all 32 PCB congeners. Marker PCB indicates the sum of the levels of PCBs 28, 52, 101, 138, 153, and 180. Dioxin-like PCB indicates the sum of the levels of PCBs 77, 81, 114, 105, 118, 126, 123, 156, 157, 169, 167, and 189.
